# Electrochemical Cathodic Polarization, a Simplified Method That Can Modified and Increase the Biological Activity of Titanium Surfaces: A Systematic Review

**DOI:** 10.1371/journal.pone.0155231

**Published:** 2016-07-21

**Authors:** Jose Carlos Bernedo Alcazar, Mabel Miluska Suca Salas, Marcus Cristian Muniz Conde, Luiz Alexandre Chisini, Flávio Fernando Demarco, Sandra Beatriz Chaves Tarquinio, Neftali Lenin Villarreal Carreño

**Affiliations:** 1 Department of Restorative Dentistry, Post-Graduate Program in Dentistry, Federal University of Pelotas, Pelotas, Rio Grande do Sul, Brazil; 2 Department of Dentistry, Science Faculty of Tocantins, Tocantins, Brazil; 3 Department of Public Health, Post-Graduate Program in Epidemiology, Federal University of Pelotas, Pelotas, Rio Grande do Sul, Brazil; 4 Department of Material Science, Post-Graduate Program in Science and Material Engineering, Federal University of Pelotas, Pelotas, Rio Grande do Sul, Brazil; University of Florida, UNITED STATES

## Abstract

**Background:**

The cathodic polarization seems to be an electrochemical method capable of modifying and coat biomolecules on titanium surfaces, improving the surface activity and promoting better biological responses.

**Objective:**

The aim of the systematic review is to assess the scientific literature to evaluate the cellular response produced by treatment of titanium surfaces by applying the cathodic polarization technique.

**Data, Sources, and Selection:**

The literature search was performed in several databases including PubMed, Web of Science, Scopus, Science Direct, Scielo and EBSCO Host, until June 2016, with no limits used. Eligibility criteria were used and quality assessment was performed following slightly modified ARRIVE and SYRCLE guidelines for cellular studies and animal research.

**Results:**

Thirteen studies accomplished the inclusion criteria and were considered in the review. The quality of reporting studies in animal models was low and for the *in vitro* studies it was high. The *in vitro* and *in vivo* results reported that the use of cathodic polarization promoted hydride surfaces, effective deposition, and adhesion of the coated biomolecules. In the experimental groups that used the electrochemical method, cellular viability, proliferation, adhesion, differentiation, or bone growth were better or comparable with the control groups.

**Conclusions:**

The use of the cathodic polarization method to modify titanium surfaces seems to be an interesting method that could produce active layers and consequently enhance cellular response, in vitro and in vivo animal model studies.

## Introduction

The use of dental implants has increased in the last decades and they are currently widely used to provide good clinical results and high survival rates of 94.4% [1, 2]. Titanium-based materials are employed for medical purposes in implants for plastic and reconstructive surgeries and orthopaedic and craniofacial reconstructions, and also in dental implantology [3].

Titanium is a highly biocompatible material, showing adequate mechanical properties, chemical stability and corrosion resistance[4]. The biocompatibility of titanium implants is attributed to the stable oxide layer[3], and together with their excellent mechanical characteristics, allow satisfactory tissue reaction, bone matrix formation, and low immune responses[1]. Even though they have a wide spectrum of advantages implant can fail, especially in patients with poor bone remnants, poor wound healing, or the presence of systemic problems such as osteoporosis and diabetes, which could reduce cellular response [5].

In order to improve the biological activity of the titanium surfaces and to promote better osteointegration and bone healing, modifications on the titanium implant surface are being used and tested, trying to preserve the titanium mechanical properties and bioinertness[6]. Electrochemical treatment is one of the surface treatments that have been indicated to be relatively simple and cheap, capable of maintaining titanium’s mechanical properties and enhancing cellular responses [6]. Anodic polarization is the electrochemical treatment widely used to deposit molecules on the surfaces, showing the improved biocompatibility of titanium due to the increasing roughness and the oxide layer of the titanium surface [7]. Cathodic polarization is an alternative of electrochemical treatment that has recently been more investigated [6]. This electrochemical treatment is a method that has been reported as a simpler method that does not need higher temperatures to be performed and can activate titanium surfaces promoting roughness and also depositing biomolecules [8]. The cathodic process could produce hydride layers, turning the possible charging of biomolecules [9], including the hydroxyapatite formation, with its capability of inducing a calcium phosphate formation in supersaturated aqueous solutions[10]. Even though the cathodic electrochemical option seems to be an interesting and simplified method to modify titanium surfaces that could enhance cellular activity and bone deposition, the literature is limited and is not consistent regarding the cellular responses.

The purpose of the present review was to systematically analyze systematically the existing studies that used the cathodic polarization technique to modify titanium surfaces to produce biological active titanium surfaces *in vitro* and *in vivo*.

## Methods

This systematic review followed the PRISMA statement and the ARRIVE statements for reporting animal researches.

Our research question was formulated using the P.I.C.O. principle to determine if the use of cathodic polarization can produce biologically active medical titanium surfaces in terms of cellular response (proliferation, adhesion, or differentiation) *in vitro* (cells)or *in vivo* (animal model).

### Search Strategy

The search was conducted in PubMed, Web of Science, Scopus, Science Direct, Scielo and EBSCOHost databases until June 2016. Google Scholar and doctoral theses related to the research questions were also searched and reviewed. No restrictions on publication data or languages were used.

Mesh terms, commonly used terms, and synonyms were included as part of the search. An extensive combination of keywords was performed, and in order to include all the studies of interest[11], final keywords used included the following terms: *("dental implants" OR implants*, *dental OR dental implant OR medical implants OR implants OR prostheses*, *surgical OR dental prosthesis*, *surgical OR surgical prostheses OR surgical dental prosthesis OR prostheses*, *surgical dental OR prosthesis*, *surgical dental OR discs) AND titanium AND (cathodic polarization OR hydride formation OR "surface deposition" OR "surface modification" OR "surface treatment")*

The sequence of the keywords were adapted according to databases requirements, for instance the Web of Science included “TS = " at the beginning of each item.

Search results were uploaded to EndNote software (version 7.0, Thompson Reuters, 1988–2013) to facilitate and standardized the literature revision and analysis.

### Study Selection

#### Inclusion criteria

Studies that used medical pure titanium or titanium implants modified by the cathodic polarization treatment, at least in one group, were included. Cathodic polarization method was considered when it was used the experimental titanium as working electrode (cathode) and a platinum electrode as anode, an acidic electrolyte solution, a controlled current density, a controlled temperature, a controlled time and galvanostatic technique.

Biological response, including cellular proliferation, adhesion, and differentiation; by *in vitro* cellular essays, animal models, or human clinical trials had to be tested to be included in our sample.

#### Exclusion criteria

Researches that evaluated titanium alloys, other material types different from titanium and orthodontic titanium appliances by mechanical or physical tests were excluded. Studies without control groups were also excluded. Modifications of the cathodic polarization standard method, literature reviews, patents, comments, editor letters, abstracts, or posters presentations were also excluded.

#### Selecting method

The selection was performed by two reviewers (MMSS and JCBA) independently and in duplicate, using the same eligibility criteria. The training and calibration process was performed prior to the formal literature analysis. For title and abstract analysis, inter-rater kappa values ranged from 0.88 to 0.93 and from 0.91 to 0.97 respectively. The selection was carried out in four stages. In a first stage, duplicated records were excluded and the titles of the remaining studies were screened to identify studies related to our research question. In the second stage, abstracts were read to localize and include studies that fulfilled the selection criteria. In the third stage, the full text was read and in the fourth stage the quality assessment was done. Differences in data extraction between the reviewers were discussed and consensus was reached.

Crosschecked bibliographies of the eligible papers for additional references were reviewed according to the eligibility criteria, and newfound studies were added if accomplished the eligibility criteria.

#### Data extraction

Data extracted were sample size; material, design and diameters of the titanium specimens; pre-treatment; studied groups; coating molecule; deposition methods used, characteristics of the cellular essay and/or animal models, cellular responses (proliferation, adhesion, and differentiation) or bone deposition as primary outcome, other mechanical or physical characterization performed, statistical analysis, and other results. Standard deviation and means were also extracted if reported.

Pre-defined data-collection worksheets were employed for the assessment of the collected records and for each selected publication. Data was organized in tables and categorized to be analyzed for systematic synthesis. Descriptive analysis (absolute and relative numbers) were performed using the software STATA 12.0 (StataCorp, College Station, TX, USA).

#### Quality assessment

Each study was evaluated according to ARRIVE and SYRCLE statements for animal model studies. The ARRIVE criteria was modified to assess the quality of the in vitro studies.

ARRIVE guidelines for reporting *in vivo* experiments in animal research present a checklist of 20 items to evaluate and have been developed using the CONSORT statement as their foundation. The SYRCLE statement had 10 items and attempted to report if the studies were low bias, high bias, or unclear bias.

## Results

**Fig 1:**
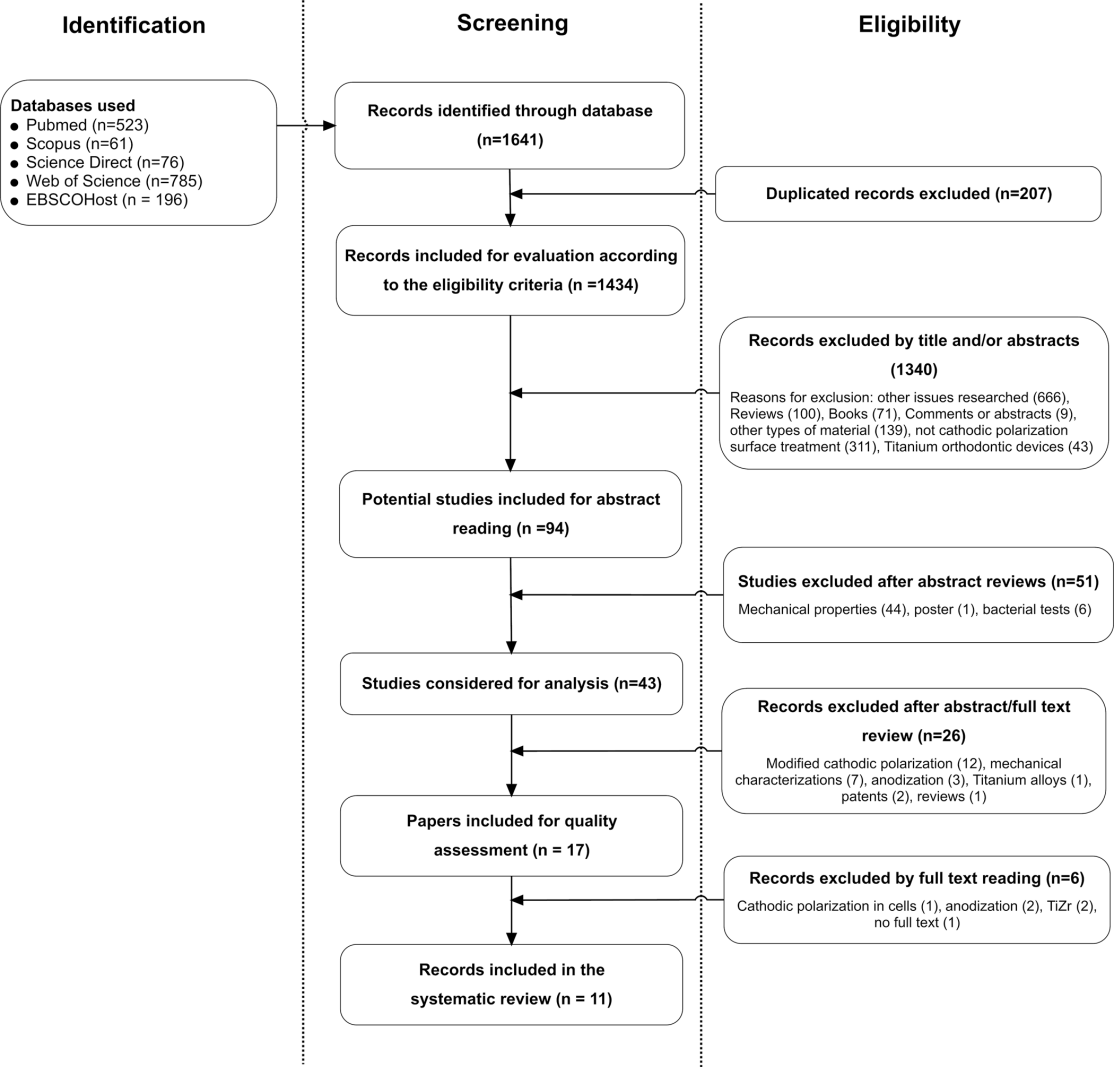
Flowchart information of the different phases of papers search and selection.

The initial search yielded 3,807 records. After the exclusion of duplicate records and the use of the eligibility criteria, 13 studies remained [9, 10, 12–22]. The identification of the papers and the selection process are presented in Fig 1. The exclusion of the studies in the last selection phase was mainly due to the use of a modified version of cathodic polarization and the used of other methods different from cathodic polarization as main outcome. Detailed reasons for exclusion are presented in S1 Table. The thirteen studies that accomplished the full eligibility criteria, were selected as final sample.

### Sensitivity analysis

Quality criteria of the studies are described in Table 1. According to the arrive criteria, 20 items were evaluated in the animal model in vivo studies. The *in vivo* animal model studies presented high or unclear risk of bias. The studies did not report information regarding selection method, sampling and allocation process, randomization, animal allocation and hostelling, blinding and dropouts, or replacement of the animals. Control of any confounders was also not reported. Overall results from seven studies with animal model showed that information was unclear (n = 15(10.7%)) or partially reported (n = 41 (29.3%)). The overall results of “No” (high risk) was 27 (21.0%), and "Yes"(low risk) was 57 (40.7%).

**Table 1:** Quality assessment according to ARRIVE and SYRCLE criteria.

Quality Criteria statements					Authors										
SYRCLE (Bias)	ARRIVE statements		Tao et al., 2016	Liang et al., 2014	Lamolle et al., 2010	Lamolle et al., 2009	Zhang et al., 2009	Young-Taeg et al., 2009	Ban et al., 1996	Frank et al., 2014	Xing et al., 2014	Huang et al., 2013	Ou et al., 2008	De Giglio et al., 2007	Hosaka et al., 2006
			AM	AM and CM	AM	AM	AM	AM	AM	CM	CM	CM	CM	CM	CM
	**1**	TITLE*	**3**	**1**	**1**	**1**	**3**	**1**	**1**	**1**	**1**	**1**	**3**	**1**	**1**
	**2**	**ABSTRACT**	**1**	**3**	**1**	**1**	**3**	**3**	**3**	**1**	**1**	**3**	**3**	**1**	**1**
		Summary of the background, research objectives*	1	1	3	3	1	3	0	1	1	0	0	1	1
		Details of the species or strain of animal used	1	0	1	1	0	3	1	4	4	4	4	4	4
		Key methods, principal findings and conclusions *	1	1	1	1	1	1	1	1	1	1	3	1	1
	**3**	**INTRODUCTION**	**1**	**1**	**3**	**3**	**1**	**1**	**3**	**1**	**1**	**1**	**3**	**1**	**1**
		Background information *	1	1	1	1	1	1	1	1	1	1	1	1	1
		Experimental approach *	1	1	1	1	1	1	1	1	1	1	3	1	1
		Relevance to human biology*	1	1	2	0	1	1	3	1	1	1	1	1	1
	4	**Objectives** *	**1**	**1**	**1**	**1**	**2**	**1**	**1**	**1**	**1**	**1**	**2**	**2**	**1**
		**METHODS**													
	**5**	**Ethical statement/ guidelines for animals care/used**	**1**	**1**	**1**	**1**	**2**	**1**	**1**	**4**	**4**	**4**	**4**	**4**	**4**
	**6**	Study design*	**2**	**2**	**2**	**2**	**3**	**2**	**2**	**2**	**2**	**2**	**2**	**2**	**2**
		Experimental and control groups*	1	1	1	1	1	1	1	1	1	1	1	3	1
**Selection**		Allocation samples*	1	0	0	0	1	0	0	0	0	0	0	0	0
**Detection**		Randomization samples*	1	0	1	0	2	0	0	0	0	0	0	0	0
**Performance/ detection**		Blinding (Researchers, caregivers, assessors)	0	0	0	0	0	0	0	0	0	0	0	0	0
	**7**	**Experimental procedure**	**3**	**1**	**3**	**3**	**3**	**3**	**3**	**1**	**1**	**1**	**1**	**1**	**1**
		in vitro cellular detail method*	4	1	4	4	4	4	4	1	1	1	1	1	1
		Specimens characteristics and preparations *	1	1	1	1	1	2	1	1	1	1	1	1	1
		Coating procedures*	1	1	1	1	1	1	1	1	1	1	1	1	1
		Anesthesia	0	1	0	0	1	1	0	4	4	4	4	4	4
		Antibiotics	0	1	0	0	0	0	0	4	4	4	4	4	4
		Analgesia	0	1	0	0	0	1	0	4	4	4	4	4	4
		Surgical procedure	1	1	1	1	1	1	1	4	4	4	4	4	4
	**8**	**Experimental animals**	3	3	1	1	3	3	3	4	4	4	4	4	4
**Selection**		Species	1	1	1	1	1	1	1	4	4	4	4	4	4
		strain, sex, developmental stage, weight	1	1	1	1	1	1	3	4	4	4	4	4	4
		source of animals	0	0	1	1	0	0	0	4	4	4	4	4	4
**Performance**	**9**	**Housing and husbandry**	**1**	**3**	**1**	**3**	**0**	**0**	**3**	**4**	**4**	**4**	**4**	**4**	**4**
		**Housing and husbandry–conditions and welfare-**	**1**	**0**	**1**	**1**	**0**	**3**	**0**	**4**	**4**	**4**	**4**	**4**	**4**
		Related assessments and interventions	1	1	1	0	0	0	1	4	4	4	4	4	4
	**10**	Sample size* **–**	**3**	**3**	**3**	**3**	**2**	**0**	**2**	**0**	**3**	**0**	**0**	**0**	**0**
		Sample size* –	1	1	1	1	2	0	2	0	1	0	0	0	0
		Sample size calculation*	0	0	0	0	0	0	0	0	0	0	0	0	0
		Number of animals in each experimental group	1	2	1	2	2	0	0	4	4	4	4	4	4
		Number of samples in each experimental group*	1	1	1	0	1	0	1	0	1	0	0	0	2
	**11**	**Allocation animals**	**3**	**0**	**3**	**0**	**2**	**0**	**0**						
		Allocation animals to experimental groups	1	0	1	0	1	0	0	4	4	4	4	4	4
		Randomization or matching	1	0	1	0	0	0	0	4	4	4	4	4	4
		Order in which animals were treated and assessed	0	0	0	0	0	0	0	4	4	4	4	4	4
	**12**	**Experimental outcomes–primary and secondary** *	**1**	**1**	**1**	**1**	**1**	**1**	**1**	**1**	**1**	**1**	**1**	**1**	**1**
	**13**	Statistical methods* **–details and unit of analysis**	**1**	**1**	**1**	**1**	**1**	**1**	**3**	**1**	**1**	**1**	**2**	**1**	**1**
	** **	**RESULTS**													
**Attrition**	**14**	**Baseline data–characteristics /health status of animals**	1	0	0	0	0	0	0	4	4	4	4	4	4
**Attrition**	**15**	**Numbers analyzed–**	3	3	3	3	3	3	3	1	1	1	1	1	1
		Absolute numbers in each group included in each analysis*	1	1	1	1	1	1	1	1	1	1	1	1	1
**Other**		Explanation for exclusion	0	0	0	0	0	0	0	4	4	4	4	4	4
**Reporting**	**16**	Outcomes and estimation–results for each analysis with a measure of precision*	**1**	**1**	**1**	**1**	**1**	**1**	**1**	**1**	**1**	**1**	**1**	**1**	**1**
**Other**	**17**	**Adverse events–details and modifications for reduction**	**0**	**0**	**0**	**0**	**0**	**0**	**0**	**4**	**4**	**4**	**4**	**4**	**4**
		DISCUSSION													
	**18**	**Interpretation**	**3**	**2**	**2**	**2**	**3**	**3**	**2**	**3**	**1**	**3**	**3**	**1**	**3**
		scientific implications*	1	1	1	1	1	1	1	1	1	1	1	1	1
		Study limitations*, bias, limitations of animal model	1	0	0	0	1	1	0	0	1	0	0	1	0
		Implications/ n animal reduction	0	0	0	0	0	0	0	4	4	4	4	4	4
	**19**	Generalisability/translation/ relevance to human biology*	**0**	**0**	**0**	**0**	**0**	**1**	**0**	**0**	**0**	**0**	**1**	**0**	**0**
	**20**	**Funding** *	**0**	**1**	**1**	**1**	**1**	**1**	**1**	**1**	**0**	**1**	**1**	**1**	**1**

* *items assess also for in vitro cellular tests* 0 = No (high risk of bias), 1 = Yes (low risk of bias) 2 = Unclear (unclear risk of bias) 3 = partial reported 4 = not applicable; AM = animal model CM = cellular model

Only 16 items from the ARRIVE criteria fit in the in vitro studies and could be evaluated. According to the criteria, in vitro cellular model studies showed medium bias. Studies presented incomplete (n = 9 (10.7%)) or unclear information (n = 9 (10.7%)). Information that accomplish "Yes" (low risk) criteria was 55 (65.5%) and "No" was 11 (13.1%) in the overall sample of in vitro studies.

Quantitative assessment according the ARRIVE criteria is presented in Table 2 and qualitative data are present in S1 Fig.

**Table 2:** Quantitative results of ARRIVE according to the 20 evaluated items.

Variables/Category	No(high)		Yes(low)		Unclear		Partial report		Total	Risk bias
	n	(%)	n	(%)	n	(%)	n	(%)		
1. Tao et al., 2016	3	15.5	9	45.0	1	5.0	7	35.0	20	Unclear/high
2. Liang et al., 2014	4	20.0	9	45.0	2	10.0	5	25.0	20	Unclear/high
3. Lamolle et al., 2010	3	15.0	10	50.0	2	10.0	5	25.0	20	Medium
4. Lamolle et al., 2009	4	20.0	9	45.0	2	10.0	5	25.0	20	Unclear/high
5. Zhang et al., 2009	4	20.0	5	25.0	4	20.0	7	35.0	20	High
6. Young-Taeg et al., 2009	5	25.0	9	45.0	1	5.0	5	25.0	20	Unclear/high
7. Ban et al., 1997	4	20.0	6	30.0	3	15.0	7	35.0	20	High
8. Frank et al., 2014*	2	14.3	10	71.4	1	7.1	1	7.1	**14**	Medium
9. Xing et al., 2014*	2	14.3	10	71.4	1	7.1	1	7.1	**14**	Medium
10. Huang et al., 2013*	2	14.3	9	64.3	1	7.1	2	14.3	**14**	Medium
11. Ou et al., 2008*	1	7.1	6	42.9	3	21.4	4	28.6	**14**	Unclear/high
12. De Giglio et al., 2007*	2	14.3	10	71.4	2	14.3	-	-	**14**	Medium
13. Hosaka et al., 2006*	2	14.3	10	71.4	1	7.1	1	7.1	**14**	Medium

* in vitro cellular test

### Data Obtained

All studies used pure titanium-grade 2 or 4 (100.0%). Pure titanium and also titanium alloys were used in two studies (15.4%). Titanium shapes were used in the studies in the form of coins (53.9%), sheets (15.4%), implants (23.1%), and bars (7.7%).

To increase the surface energy, eleven studies (84.6%) prepared the experimental titanium surfaces by grinding, polishing, etching, washing, and drying[9, 10, 13–19, 21, 22]. Cathodic polarization technique was similar in all studies. It was used platinum as anode, the titanium sample as cathode and the control electrode was calomel (SCE) or silver. The technique included in all cases, the use of acidic solutions as a conduction medium.

The main results of the studies are presented in Table 3. Cathodic polarization was mainly used to coat biomolecules in eight [9, 10, 12, 14, 15, 17, 20, 21]studies (61.6%) three studies (23.1%) used the cathodic polarization for hydration of the titanium surfaces[16, 18, 22], and two studies (15.4%) used acid electrolytes[13, 19]. The deposited biomolecules included calcium phosphate derivate in five studies (38.5%), such as hydroxyapatite and brushite. Other molecules such as enamel matrix derivate, Magnesium, Strontium were also used in three studies (23.1%). Acids such as Pyrrole-3-acetic modified, oxalic acetic or tartaric acids were used to promote surface modifications in five investigations (38.5%).

**Table 3:** Surface properties and cellular responses from in vitro and in vivo-animal model studies.

AUTORS	EXPERIMENTAL PROCEDURE	SURFACE CHEMISTRY AFTER TREATMENT		SURFACE MORPHOLOGY OR FILM COMPOSITION	STRENGHT RESISTENCE: PULL-OUT, REMOVAL TORQUE, STABILITY.	BONE GROWN /CELL ADHESION
*IN VIVO-ANIMAL MODEL*						
Ban et al. 1997	Hydroxyapatite (HA)deposition	Basic elements of Apatite were present on the titanium surface.	HA surface only had Spherical particles of HA. Surface with HA electrochemically coated is covered by needle-like precipitates.		Higher strength resistance from pull-out test was observed in the titanium treated by electrochemical methods than the control samples, after 3 and 6 weeks of implantation	The formation of new bone was enhanced in the electrochemical treated surface compared to the control surfaces.
Lamolle et al., 2009	Hydrofluoric acid (HF)	Hydride, Fluoride and Oxide were present.	The oxide concentration was higher in the 0.001% HF at 30nm. Low concentration of HF increased hydrophobicity.		Implants modified by cathodic reduction with 0.01 vol % hydrofluoric acid showed the highest pull out strength (p< 0.05) followed by the 0.1vol%.	The concentrations of the fluoride and hydride in the titanium implant modified surfaces was correlated to the in vivo bone retention(r = 0.94).
Lamolle et al., 2010	Hydrofluoric acid (HF)	Hydride, Fluoride and Oxide were present.	Groups of implants with 0.001% and 0.01% HF showed the highest fluoride content at their surface structure		-	All experiment groups showed new peri-implant cortical bone, but implants treated with 0.01% HF showed higher osteocalcin, collagen-I and TRAP, revealing an advanced osseointegration process. Implants modified with 0.001% and 0.01% HF presented a statistically significant increased newly formed bone. Lower presence of blood was observed at the interface after removal of the implant in the groups of implants (0.001% and 0.01% HF). The control group scored higher LDH activity than all the test groups.
Liang et al., 2014	Pure brushite and modified brushite with 5%, 10%, and 20% Strontium (Sr) deposition	Basic elements of Brushite and Strontium were present on the titanium surface.	Brushite coating, presence of crystals, some arranged in clusters. Brushite coating containing 20% Sr showed an irregular surface morphology		Removal torque strength in 5% Sr and 10% Sr groups was significantly increased compared with the other three groups without cathodic treatment (p < 0.01)	After 24, 48, or 72h the number of the proliferating cells on the brushite-coated and Sr-doped brushite groups were higher than in the control group (p < 0.01), especially in the 10% Sr-doped coating. Modified surfaces with 5% and 10% Sr-doped brushite coatings were associated with increased 3D bone volume(p < 0.05), especially around the 10% Sr-doped brushite-coated implants.
Tao et al., 2016	Electrochemical deposition of Zinc(Zn), Strontium, Magnesium(Mg), and HA.	Coatings composed of hydroxyapatite containing 10% Zn, Mg and Sr ions on titanium.	-		Push-out force of group Sr-HA was significantly higher than that of groups Zn-HA and Mg-HA. Group Sr-HA showed the strongest effects on all micro-CT parameters (bone volume, trabecular thickness, connective density, trabecular number; trabecular separation) significantly (p<0.05).	After 12 weeks, new bone was formed. Within the circumference of marrow cavities of cortical bone, there were osteoblast-like cells, suggesting the beginning of new bone formation. There was more bone tissue on implant surfaces of Zn-HA, Mg-HA, Sr-HA-coated implants than in those of HA-coated.
Young et al.,2009	Electrochemical deposition of Magnesium, Phosphate	Magnesium, P, and Ti were identified in the composition.	The implants had moderate roughness of 0.7–1.4 mm. Oxidized implants had crystal structures consisting of a mixture of Anatase and rutile phase.		After 6 weeks of healing, all surfaces increased implant stability but it was higher in the modified surfaces than in the control surfaces.	New bone formation occurred in all surfaces, but it was increased in the Mg-MP implant group.
Zhang et al., 2009	Solution of calcium phosphates as medium	Titanium hydride was identified. Calcium and Phosphates were present on the titanium surface.	Metal surface were rough and had fine granular appearance. A thin layer of CaP (100nm thick) was deposited and had higher resistance to displacement.		**-**	Bone growth was fast in the electrochemical-treated specimens. After 4weeks bone formation and the amount of bone in electrochemical titanium and stainless steel samples were significantly higher than that in control without cathodic treatment (p < 0.01).
**IN VITRO STUDIES**						
Franck et al., 2014	Enamel Matrix Derivate (EMD) deposition	EMD was coated. Characteristic elements were identified in the composition.	Electrochemical EMD coated samples presented larger spherical structures attached to the surface. Sandblasted and acid-etched revealed nano-nodules and small spherical structures on the surface.		**-**	No cytotoxicity was observed in any group. For Electrochemical treated groups the expression of Coll-1 mRNA levels and the alkaline phosphatase activity was significantly higher compared to control.
Xing et al., 2014	Acids (oxalic: OA)as medium	Presence of Hydride. Characteristic elements were identified in the composition.	OA created the roughest surface and thin layers.		**-**	At day 3, cells grown in all groups faster than in the control. The proliferation rate on acetic acid was significantly higher than others groups. Hydrogen amount on the surfaces was correlated with proliferation rate at day 3 (r 5 0.973, p <0.05). At day 6, proliferation of cells was higher in tartaric and control groups only.
Huang et al., 2013	Hydroxyapatite (HA) deposition	Deposition of HA. HA surface was mainly composed of O, P, Ca, and Si. Si content was 7.77 wt.%±0.39.	HA and HA/CS films formed uniform layers on the Ti substrate. The HA/CS coating had a porous structure and the HA coating had a dense surface structure.		**-**	After 7 days, cell proliferation on the HA/Cathodic coated surfaces was higher (p < 0.05) than those on HA coating.
Ou et al., 2008	Electrochemical treatment	Promote the presence of Hydride. O_2_ concentration following electrochemical treatment was higher than in polished Ti.	Titania film with cathodic pre-treatment and anodization was thicker than other groups Porosity was higher in ACTi samples		**-**	Cathodic pre-treatment followed by anodization at 24h significantly more cells attached than controls (cathodic and anodization treatment only). Cells on AC-Ti were more spread out and had more, longer filopods than other groups.
De Giglio et al., 2007	Pyrrole-3-acetic acid, 4-fluoro-phenylalanine deposition	Promote deposition of 4-fluoro-phenylalanine modified PPy-3-acetic film.				Cell Adhesion, growth, and viability of osteoblast-like cells onto PPy-3-acetic modified titanium substrates were comparable to the control groups. Cell phenotype was similar in all groups.

Titanium characterization was performed in all studies. The tests included surface morphology by scanning electron microscopy (SEM) or transmission electron microscopy (TEM);surface chemistry by X-ray photoelectron spectroscopy (XPS) or secondary ion mass spectrometry(SIMS),blue light laser profilometer, UV–vis spectroscopy, field emission scanning electron microscopy(FE-SEM), X-ray diffraction, and contact angle measurement.

Biocompatibility and cellular response tests performed were cytotoxicity, cell proliferation, adhesion and differentiation, cell morphology observation, and RNA isolation-reverse transcriptase (RT)-PCR amplification. For bone growth assessment, Rx and bone mineral density, micro-computed tomography (Micro-CT), confocal laser microscopy, osseointegration histological analysis, and pull-out tests were conducted.

Studies performed *in vitro* cellular tests (46.2%), six studies opted by the *in vivo* animal model (46.2%) and one study (7.7%) was performed in vitro and in vivo essay.

The cellular types used were MC3T3-E1osteoblast-like cells (71.4%), human gingival fibroblast (14.3%), NIH3T3, and fibroblasts (14.3%).

Studies showed that the cathodic polarization method promoted incorporation of the biomolecules, such as phosphate hydroxyapatite derivate, antibiotics, and enamel matrix derivate, on the titanium surfaces.

*In vitro* cellular tests showed significantly higher cellular proliferation [13, 15, 19]or adhesion[13, 14, 18] after 3–7 days in the experimental (hydrided or coated) groups than in control groups. Cytotoxicity was similar in all groups [19]and gene expression of Coll-1mRNA and alkaline phosphatase was increased in the coating groups compared to the control groups [9].

*In vivo* animal model studies reported no toxic effect enhancement on bone formation[12, 17, 21], showing balance in gene expression between some biological factors such as osteocalcin, collagen-I or TRAP[22], and bone retention [16]in the groups of titanium hydrided or with biomolecule coatings[10, 20] compared to control groups after 3–12 weeks of experiment.

## Discussion

To the best of our knowledge, this review is the first to systematically collect the existing evidence in relation to the cell response to the titanium surfaces modified by the cathodic polarization technique

Indeed, there are several different techniques of deposition to modify medical titanium surfaces including plasma, sputter-deposition, sol–gel coatings, electrochemical deposition, or biomimetic precipitation. Plasma-spraying is widely used [23], as well as the anodic method to coat molecules. Even though there are advantages of these last two techniques, they present some drawbacks, such as low adhesion and thicker coating, respectively. These problems were attributed to technique issues produced essentially by the temperature used.

Electrodeposition using titanium as a cathode is usually conducted in acidic electrolytes, organic or inorganic, in order to modify surfaces of titanium for hydridation or deposition of molecules. The techniques make it possible to control the thickness of the coating deposit on all kinds of surfaces and reduce the time required for coating, as the process is highly reproducible and efficient[12].

Results from our included studies indicated that the use of the cathodic polarization as surface treatment in acid solutions induced the hydride layers’ formation on titanium, increasing positive cellular responses regarding proliferation, adhesion, and differentiation [16, 21, 22].Other studies that used this technique reported the reduction of the mechanical properties of titanium due to hydrogen embrittlement produced by the hydride layers, which could possible cause implant fractures [24, 25]. Recent studies showed that the presence of the hydride layer obtained using cathodic polarization offers the potential for attaching biomolecules, such as antibiotics and hydroxyapatites in ambient temperatures, on pure titanium, titanium alloys [9], or other metal types. In fact, this has been the main advantage of the cathodic polarization so far[12].

In the present review, most of the investigation have deposited biomolecules on titanium and they observed positive deposition in terms of integrity, adhesion and thickness, also detecting enhanced cell adhesion. Biomolecules deposited on the surface were observed to maintain their integrity, presenting good interaction with the metal surface and having binding strength[17]. The layers generated were mostly thin (100um min) but dense enough to promote good strength of the coatings [10].

Bioactive molecules can be adsorbed, affecting cell attachment to the surface and tissue response. Several molecules could be deposited to improve titanium surfaces in terms of biological responses such as magnesium, strontium, or bone proteins [9, 20, 26]. For instance, the electrochemical deposition of calcium-phosphate-derivate molecules can increase fixation of implants to bone tissue, promoting better adhesion and activation of bone cells on the implant surfaces[12]. Another advantage is the possibility to deposit drugs on the active surfaces, which could be released over a period of time, enhancing the cellular responses [27].

Some factors related to the method of the cathodic technique deposition can influence the film characteristics and consequently cellular response[6]. In this review, the eligibility criteria attempted to include studies presenting similar characteristics regarding the cathodic method used.

Most of the studies used room temperatures to performed the test in the range of 20°C to 25°C. The technique of deposition is performed at ambient temperatures, being that such characteristics are probably responsible for the good conformability to the shape of the molecules’ components on titanium surfaces, the thickness of the films (less than 1 µm), and the increased resistance to delamination for the coating homogeneity and the stronger adhesion of the coating[6]. Lower temperatures during deposition of the film can decrease the presence of defects or pores on hidroxyapatite crystals [28].

Cathodic polarization in acid can optimize titanium implant surfaces for improved osseointegration. In the present review, most of the studies opted to use electrolytes with lower pH (2–6). It was reported that baths with pH of 4.11 produce the deposition of hydroxyapatite films on titanium alloy[29]. Studies have observed that increasing the pH to 5 [30]or nearly neutral (7.2)[31] produce mono grain phases. Hydrofluoric acid has been shown to increase the hydride and fluoride amount on Ti surfaces, changing the porosity of the surface and consequently the surface roughness[19]. These alterations were positively correlated within vivo bone retention and peri-implant bone mineralization [16]

Cell adhesion, growth, and biocompatibility between osteoblast-like cells and treated surfaces have increased, preserving osteoblastic phenotype [13]. The expression of proteins such as Coll-1 mRNA and alkaline phosphatase activity indicated the bone proliferation activity promoted by the modified surface [9].

The current density is another factor that can influence the films in relation to mechanical, physical and biological characteristics. In our review, studies used current densities in the range of 0.4-20mA/cm2 or -2.0–2.5V during 30 to 60 minutes. The density current determinates the format and the adhesion of the deposit. It has been shown that decreasing the current density produced deposits with needle forms and increasing it can produce blunt forms of the particles of hydroxiapatite[32]. When associate with lower pH and/or with stirrings—ultrasonic or magnetic- the size of the particle could be reduced. The size of the particle is also important since smaller particles were found to enhance the cellular adhesion on the surface [29]. On the other hand, lower current densities in the range of 0.2-15mA/cm^2^ increased bond strengths of the coating [33].

In our study, the sample was composed of investigation using galvanostatic technique. The galvanostatic technique allowed to work in acid or near physiological pH at body temperature and doesn`t require post-treatment, which is required in the pulse electrochemical method where a post-treatment at high temperatures in the range of 300°C-800°C is needed. Higher temperatures could negatively have affected the mechanical properties of the coated surface as aforementioned.

The studies included were composed of in vitro cellular essays and in vivo animal model experiments that used cathodic polarization method to modified titanium surfaces. In vitro results reported significant higher cellular proliferation, gene expression of bone formation genes and low cytotoxicity. Animal experiments reported no toxic effect, enhancement of bone formation and bone retention in the groups with modified surface of titanium by the cathodic technique. In fact, all the studies with animal models were performed after an in vitro essay. For instance, in vitro observation of titanium surfaces showed needle-like carbonate apatite, this new composite enhance the mechanical bonding strength in early stages of implantation in the animal model, increasing the filling of the gap between the implant and the surface with new bone compared to those without the treatment[12]. The surface modifications, in macro and micro level can efficiently increase biological events in vitro and in vivo[8]. In the study of Young-Taeg et al., 2009, the treatments performed on titanium modified the topographies regarding the roughness and also produce chemistry changes on surfaces. These changes were associated with improvement of osseointegration since the oxidized groups by cathodic polarization presented better stability and bone density[20]. The author also sustained that when implants stability in early stages is low, subsequent resonance values increased rapidly over the time, possibly due to difference in bone properties between the animals and humans[20].

The hydrophilicity is an important factor in the enhancement of the bone response [6, 8, 23]. Hydrophilicity was demonstrated to increased osseointegration when using in vitro and in vivo animal models [34]. This situation was also detected by Lamolle et al., 2009, where a positive correlation was found between high bone retention and high amounts of Fluoride and the hydride in the surface in the group with lower concentration of electrolyte. The author explained that this could be due to the aggressive conditions produced by the high electrolyte concentration, once at low hydrofluoric acid concentrations the surfaces were weakly etched, and consequently, kept higher amounts of fluoride, oxide, and hydride.Also a correlation was observed between some roughness parameters regarding positive surface skewness that means elevations on flat surfaces, kurtosis higher than 3 that is rounded peaks and high core fluid retention (more spaces between the peaks) with high bone retention of implants. Studies showed the importance of the roughness and its relation with higher bond strength [35] and the before mention study showed that a conjunct of surface parameters can predict the in vivo performance of bone retention[16].

It is clear that in vitro controlled methods allowed to obtain more objective results, nevertheless is difficult to extrapolated such findings directly to the in vivo animal models, since several factors cannot be controlled in animal experimentation as opposite to the in vitro condition[36]. The strengths of the animal experiments include the possibility of learning about some biological mechanism in an living organism, turning their results more representative to the clinical situation than those obtained in vitro [37].

It was suggested that systematic reviews and meta-analyses on animal experiments can be conducted in order to model relevant clinical problems since some treatments are currently being offered to vulnerable groups of patients without much evidence of their beneficial effects [38, 39].

Some limitations should be considered. We did not find any in vivo clinical trials using prosthetic appliances obtained by cathodic polarization, making the clinical effect of this technique unclear. Additionally, systematic reviews including animal experiments or cellular responses are different situations from the in vivo clinical reality; however, it allows a more objective appraisal of the research evidence from the traditional narrative reviews, and also offer a sensible and rational approach to assessing the translational potential of promising experimental interventions before decisions are made to proceed with clinical trials [38, 39].

Our findings should be considered with caution, since according to the ARRIVE quality criteria the studies with animal experiments showed medium or high risk of bias due to the incomplete or lack of data reported. It has been discussed the importance to report information such as the study design, animal characteristics, housing and husbandry, allocation of the animal, eventual exclusions or adverse events in the studies with animal models, which could influence the results, especially when is testing new medicines or drugs[37, 40]. However, since there is no evidence testing titanium surfaces modified by cathodic polarization in human clinical trials, the results obtained from our sample of studies using animal experiments can generate contribution firstable with the necessity and importance of a good quality reporting of results when an animal model study is performed, and second, because of the clear necessity of more evidence using animal model before thinking in human clinical trials.

This systematic review showed that the use of cathodic polarization produced adequate cellular response promoting proliferation, differentiation, and bone development in vitro and in animal experiments. The cathodic polarization seems to be a feasible alternative to successfully modified surfaces, maintaining adequate mechanical and biologic properties, allowing deposit of biomolecules and promoting activity on the surface by hydrided formation.

## Conclusion

Cathodic polarization promotes titanium surface modifications, increasing the adhesion of active biomolecules and hydridation of titanium surfaces. Modified surfaces enhance cellular response in vitro and in vivo-animal models.

## Supporting Information

S1 PRISMA Checklist PRISMA Checklist. (DOCX)

S1 Fig Figure presenting the quantitative results means of the ARRIVE criteria for in vitro and animal model studies. (TIF)

S1 Table Excluded studies and reasons for exclusion. (DOCX)
