# Supplementary figures and images for: Electrochemical Cathodic Polarization, a Simplified Method That Can Modified and Increase the Biological Activity of Titanium Surfaces: A Systematic Review
Source: PLoS One. 2016 Jul 21;11(7):e0155231. doi: 10.1371/journal.pone.0155231 (PMC4956102; doi:10.1371/journal.pone.0155231)

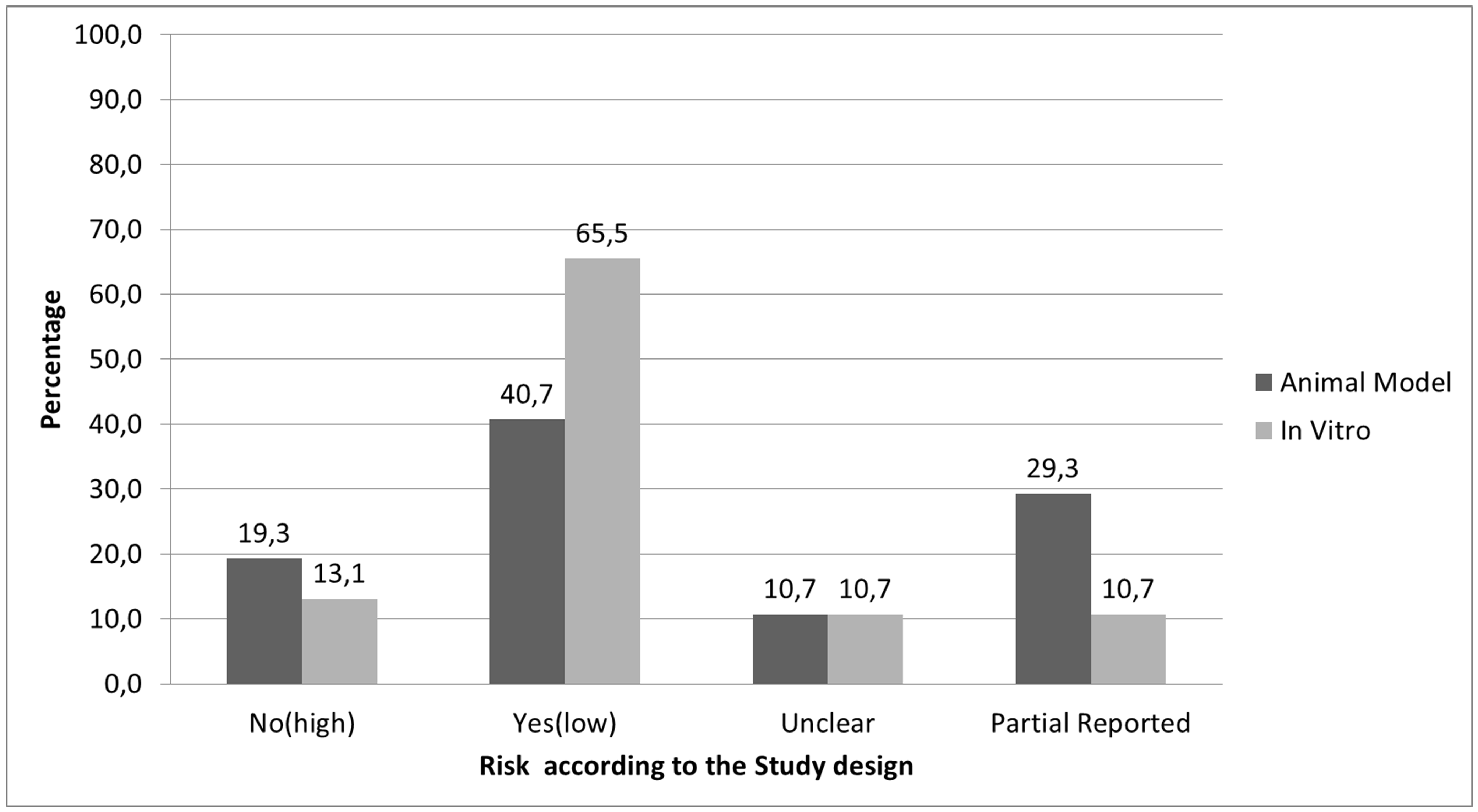

Supplement: S1 Fig — (TIF) [file pone.0155231.s002.tif]
