# Supplementary material for: Electrochemical Cathodic Polarization, a Simplified Method That Can Modified and Increase the Biological Activity of Titanium Surfaces: A Systematic Review
Source: PLoS One. 2016 Jul 21;11(7):e0155231. doi: 10.1371/journal.pone.0155231 (PMC4956102; doi:10.1371/journal.pone.0155231)
Supplement: S1 Table — (DOCX) [file pone.0155231.s003.docx]

**S1 Table.** Excluded studies and reasons for exclusion

| **Authors** | **Reasons** |
| --- | --- |
| (Burgos-Asperilla, Fierro et al. 2015; Ehrensberger et al., 2015; Ehrensberger, Sivan et al. 2010; Kalbacova, Roessler et al. 2007) | Cathodic polarization in cells |
| (Alves, Ginani et al. 2011, Strąkowska et al., 2016; Tao et al., 2016; Zhang et al., 2015; Zhang and Leng, 2005) | Cathodic plane nitriding plasma |
| (Kim et al. 2011, Frank, Walter et al. 2013, Wolf-Brandstetter, Oswald et al. 2014) | Cathodic polarization without biological tests |
| (Cheng, Lee et al. 2006; Cheng, Lee et al. 2007; Kuo et al., 2012) | Cathodic polarization as pre-treatment |
| (Lai et al., 2011; Liang et al., 2015; Ordikhani et al., 2014; Wang, Sun et al. 2012; Wang, Sun et al. 2013) | Cathodic arc plasma |
| (Gugelmin et al., 2015, Kazek-Kesik, Krok-Borkowicz et al. 2014, Kohal, Wolkewitz et al. 2009; Szabó et al., 1999; Swanson, Cheng et al. 2011; Yu et al., 2015) | Anodic polarization |
| (Etminanfar et al., 2016; Ferraris et al., 2015; Gomez-Florit, Xing et al. 2014; Song et al., 2015; Walter, Frank et al. 2014; Zhao et al., 2016) | Titanium alloys |
| (Ou et al., 2015) | Unclear electrochemical treatment |
| (Alfarsi et al., 2015; Bhattarai et al., 2015; Cho et al., 2016; Chouirfa et al., 2016; Ghimire et al., 2015; Hegedus et al., 2016; Huang et al., 2013; Huang et al., 2015; Kim et al., 2016; Kwon et al., 2015; Lee et al., 2016; Malekzadeh et al., 2016; Ogawa et al., 2016; Säuberlich, Klee et al. 1999, Sevilla et al., 2016; Sharma et al., 2016; Tack et al., 2015; Tsou et al., 2015; van Oirschot et al., 2016; Wennerberg, Fröjd et al. 2011, Yang et al., 2015) | Other surface treatments (Grinding, dip coating, SLA, hidrothermal) |

Alfarsi MA, Hamlet SM, Ivanovski S: The effect of platelet proteins released in response to titanium implant surfaces on macrophage pro-inflammatory cytokine gene expression. Clinical implant dentistry and related research 2015;17:1036-1047.

Bhattarai G, Lee Y-H, Lee M-H, Park I-S, Yi H-K: Insulin-like growth factor binding protein-3 affects osteogenic efficacy on dental implants in rat mandible. Materials science & engineering C, Materials for biological applications 2015;55:490-496.

Cho Y-D, Kim S-J, Bae H-S, Yoon W-J, Kim K-H, Ryoo H-M, Seol Y-J, Lee Y-M, Rhyu I-C, Ku Y: Biomimetic approach to stimulate osteogenesis on titanium implant surfaces using fibronectin derived oligopeptide. Current pharmaceutical design 2016.

Chouirfa H, Migonney V, Falentin-Daudre C: Grafting bioactive polymers onto titanium implants by uv irradiation. RSC advances 2016;6:13766-13771.

Ehrensberger MT, Tobias ME, Nodzo SR, Hansen LA, Luke-Marshall NR, Cole RF, Wild LM, Campagnari AA: Cathodic voltage-controlled electrical stimulation of titanium implants as treatment for methicillin-resistant staphylococcus aureus periprosthetic infections. Biomaterials 2015;41:97-105.

Etminanfar MR, Khalil-Allafi J, Montaseri A, Vatankhah-Barenji R: Endothelialization and the bioactivity of ca-p coatings of different ca/p stoichiometry electrodeposited on the nitinol superelastic alloy. Materials science & engineering C, Materials for biological applications 2016;62:28-35.

Ferraris S, Bobbio A, Miola M, Spriano S: Micro- and nano-textured, hydrophilic and bioactive titanium dental implants. Surface & Coatings Technology 2015;276:374-383.

Ghimire N, Foss BL, Sun Y, Deng Y: Interactions among osteoblastic cells, staphylococcus aureus and chitosan-immobilized titanium implants in a post-operative co-culture system: An in vitro study. Journal of biomedical materials research Part A 2015.

Gugelmin BS, Santos LS, Ponte HD, Marino CEB: Electrochemical stability and bioactivity evaluation of ti6al4v surface coated with thin oxide by eis for biomedical applications. Materials Research-Ibero-American Journal of Materials 2015;18:602-607.

Hegedus C, Ho CC, Csik A, Biri S, Ding SJ: Enhanced physicochemical and biological properties of ion-implanted titanium using electron cyclotron resonance ion sources. Materials 2016;9.

Huang M-S, Chen L-K, Ou K-L, Cheng H-Y, Wang C-S: Rapid osseointegration of titanium implant with innovative nanoporous surface modification: Animal model and clinical trial. Implant dentistry 2015;24:441-447.

Kim HS, Kim YJ, Jang JH, Park JW: Surface engineering of nanostructured titanium implants with bioactive ions. Journal of dental research 2016.

Kuo PCH, Chou HH, Lin YH, Peng PW, Ou KL, Lee WR: Effects of surface functionalization on the nanostructure and biomechanical properties of binary titanium-niobium alloys. Journal of the Electrochemical Society 2012;159:E103-E107.

Kwon Y-D, Yang DH, Lee D-W: A titanium surface-modified with nano-sized hydroxyapatite and simvastatin enhances bone formation and osseintegration. Journal of biomedical nanotechnology 2015;11:1007-1015.

Lai CH, Chang YY, Huang HL, Kao HY: Characterization and antibacterial performance of zrcn/amorphous carbon coatings deposited on titanium implants. Thin Solid Films 2011;520:1525-1531.

Lee C-H, Kim Y-J, Jang J-H, Park J-W: Modulating macrophage polarization with divalent cations in nanostructured titanium implant surfaces. Nanotechnology 2016;27:085101-085101.

Liang Y, Xu J, Chen J, Qi M, Xie X, Hu M: Zinc ion implantation‑deposition technique improves the osteoblast biocompatibility of titanium surfaces. Molecular medicine reports 2015;11:4225-4231.

Malekzadeh BÖ, Ransjo M, Tengvall P, Mladenovic Z, Westerlund A: Insulin released from titanium discs with insulin coatings-kinetics and biological activity. Journal of biomedical materials research Part B, Applied biomaterials 2016.

Ogawa ES, Matos AO, Beline T, Marques ISV, Sukotjo C, Mathew MT, Rangel EC, Cruz NC, Mesquita MF, Consani RX, Barão VAR: Surface-treated commercially pure titanium for biomedical applications: Electrochemical, structural, mechanical and chemical characterizations. Materials Science and Engineering: C 2016;65:251-261.

Ordikhani F, Tamjid E, Simchi A: Characterization and antibacterial performance of electrodeposited chitosan-vancomycin composite coatings for prevention of implant-associated infections. Materials science & engineering C, Materials for biological applications 2014;41:240-248.

Ou K-L, Hsu H-J, Yang T-S, Lin Y-H, Chen C-S, Peng P-W: Osseointegration of titanium implants with slaffinity treatment: A histological and biomechanical study in miniature pigs. Clinical oral investigations 2015.

Sevilla P, Vining KV, Dotor J, Rodriguez D, Gil FJ, Aparicio C: Surface immobilization and bioactivity of tgf-1 inhibitor peptides for bone implant applications. Journal of Biomedical Materials Research Part B-Applied Biomaterials 2016;104:385-394.

Sharma S, Bano S, Ghosh AS, Mandal M, Kim H-W, Dey T, Kundu SC: Silk fibroin nanoparticles support in vitro sustained antibiotic release and osteogenesis on titanium surface. Nanomedicine: Nanotechnology, Biology, And Medicine 2016;12:1193-1204.

Song W, Song X, Yang CX, Gao S, Klausen LH, Zhang YM, Dong MD, Kjems J: Chitosan/sirna functionalized titanium surface via a layer-by-layer approach for in vitro sustained gene silencing and osteogenic promotion. International journal of nanomedicine 2015;10:2335-2346.

Strąkowska P, Beutner R, Gnyba M, Zielinski A, Scharnweber D: Electrochemically assisted deposition of hydroxyapatite on ti6al4v substrates covered by cvd diamond films - coating characterization and first cell biological results. Materials science & engineering C, Materials for biological applications 2016;59:624-635.

Szabó G, Kovács L, Vargha K, Barabás J, Németh Z: A new advanced surface modification technique--titanium oxide ceramic surface implants: The background and long-term results. Journal of long-term effects of medical implants 1999;9:247-259.

Tack L, Schickle K, Böke F, Fischer H: Immobilization of specific proteins to titanium surface using self-assembled monolayer technique. Dental Materials: Official Publication Of The Academy Of Dental Materials 2015;31:1169-1179.

Tao ZS, Zhou WS, He XW, Liu W, Bai BL, Zhou Q, Huang ZL, Tu KK, Li H, Sun T, Lv YX, Cui W, Yang L: A comparative study of zinc, magnesium, strontium-incorporated hydroxyapatite-coated titanium implants for osseointegration of osteopenic rats. Materials science & engineering C, Materials for biological applications 2016;62:226-232.

Tsou H-K, Chi M-H, Hung Y-W, Chung C-J, He J-L: In vivo osseointegration performance of titanium dioxide coating modified polyetheretherketone using arc ion plating for spinal implant application. BioMed research international 2015;2015:1-9.

van Oirschot BAJA, Eman RM, Habibovic P, Leeuwenburgh SCG, Tahmasebi Z, Weinans H, Alblas J, Meijer GJ, Jansen JA, van den Beucken JJJP: Osteophilic properties of bone implant surface modifications in a cassette model on a decorticated goat spinal transverse process. Acta biomaterialia 2016;37:195-205.

Yang DH, Lee D-W, Kwon Y-D, Kim HJ, Chun HJ, Jang JW, Khang G: Surface modification of titanium with hydroxyapatite-heparin-bmp-2 enhances the efficacy of bone formation and osseointegration in vitro and in vivo. Journal of tissue engineering and regenerative medicine 2015;9:1067-1077.

Yu F, Addison O, Baker SJ, Davenport AJ: Lipopolysaccharide inhibits or accelerates biomedical titanium corrosion depending on environmental acidity. International Journal of Oral Science 2015;7:179-186.

Zhang M, Ma SL, Xu KW, Chu PK: Corrosion resistance of praseodymium-ion-implanted tin coatings in blood and cytocompatibility with vascular endothelial cells. Vacuum 2015;117:73-80.

Zhang QY, Leng Y: Electrochemical activation of titanium for biomimetic coating of calcium phosphate. Biomaterials 2005;26:3853-3859.

Zhao JM, Hwang KH, Choi WS, Shin SJ, Lee JK: Biological behavior of osteoblast cell and apatite forming ability of the surface modified ti alloys. Journal of nanoscience and nanotechnology 2016;16:1541-1544.
